# Treatment Related Hematologic Changes in a Population of Iranian Patients with Chronic Hepatitis C Infection from 2009 to 2014

**Published:** 2017-10

**Authors:** Zahra AHMADINEJAD, Zahra ABDILIAEI, Rizan MOHAMADI, Omid REZAHOSSEINI

**Affiliations:** 1. Dept. of Infectious Diseases, Imam Khomeini Hospital Complex, Tehran University of Medical Sciences, Tehran, Iran; 2. Dept. of Cardiology, School of Medicine, Tehran University of Medical Sciences, Tehran, Iran

**Keywords:** Interferon, Ribavirin, Leukopenia, Anemia, Thrombocytopenia, Hepatitis C, Genotype

## Abstract

**Background:**

We aimed to assess the frequency of hematologic changes, response to treatment, rate of discontinuation and dose reduction in Hepatitis C positive patients, treated with Interferon and ribavirin from Apr 2009 to Mar 2014.

**Methods:**

In this cross-sectional study, out of registered patients, 554 were assessed and 150 patients with positive HCV PCR, regular patient visits to clinic and complete records were included. HCV viral load, complete blood count and liver enzyme levels were measured before initiation of treatment and monthly. Exclusion criteria's were other types of hepatitis and HIV infection, autoimmune or blood diseases, illegal drug use and treatment with bone marrow suppressors. The data was analyzed using SPSS.

**Results:**

Out of 150 patients, 135 (90%) were male. Mean age was 39.7±10.7 (range 23–74) yr old. Forty-six patients (30.7%) had genotype 1 and 99 (66%) genotypes 2 and 3. Treatment regimens were prescribed as Pegafron+Ribavirin in 125 (83.3%), Interfron+Ribavirin 9 (6%) and Pegintron+Ribavirin in 16 (10.7%) of patients... The prevalence of anemia in genotype 1 patients was higher (*P*=0.044). There was no association between sex and leukocyte changes. Thirty-four (22.7%) patients had moderate and severe thrombocytopenia. Six patients had severe anemia and Ribavirin dose was adjusted.

**Conclusion:**

The hematological changes are common side effects of conventional hepatitis C treatment regiments. Although drug dose adjustment is not usually necessary, due to severe anemia in genotype 1 patients, we should treat high-risk patients cautiously and made the appropriate changes in drug dosage at the right time.

## Introduction

Hepatitis C is a chronic inflammatory disease of the liver caused by hepatitis C virus (HCV), an RNA virus and a member of the family flaviviridae. The most common route of transmission of this disease is via contaminated blood (1).

Currently, about 170 million people are infected with HCV worldwide (2). Hepatitis C is the most common cause of chronic liver diseases after alcoholic hepatitis. Number of people with positive hepatitis C is less than 1% in different cities of Iran (1). The disease is chronic in the majority of patients and leads to cirrhosis and liver cancer and the patient needs to liver transplantation (1, 3). Hence, treatment of hepatitis C and prevention of progress to cirrhosis is important and eradication of HCV is the goal of the treatment.

During the past decade, there has been great improvement in the treatment of HCV-positive patients. Available treatments for hepatitis C in Iran are combination of conventional or pegylated interferon (PEG-IFN) and ribavirin (RBV) for 24 to 48 wk, according to virus genotype, which resulted in about 50% SVR (Sustained virologic response) for genotype 1 and 80% for genotype 2 and 3 (1, 4).

Two types of interferon are approved for treatment of hepatitis C includes PEG-IFN alfa-2a (Pegasys or Pegaferon) and peg-IFN alfa-2b (Pegintron). Standard duration of treatment for PEG IFN plus RBV is about 48 wk for genotype 1 or 4 and 24 wk for genotype 2 or 3.

Combination therapy can always be accompanied by a series of adverse effects, including Flu like syndrome, depression, autoimmune disorders, and hematologic adverse effects such as anemia, neutropenia, and thrombocytopenia that occur in about 32% of patients (5).

Hematologic adverse effects are well tolerated in most cases and only in rare cases lead to a dose reduction. In this case, there is no accurate data and statistics. In this study, we aimed to assess the frequency of hematologic changes and the effects of them on the response to treatment, and discontinuation or dose reduction of treatment regimens.

## Materials and Methods

**Fig. 1:**
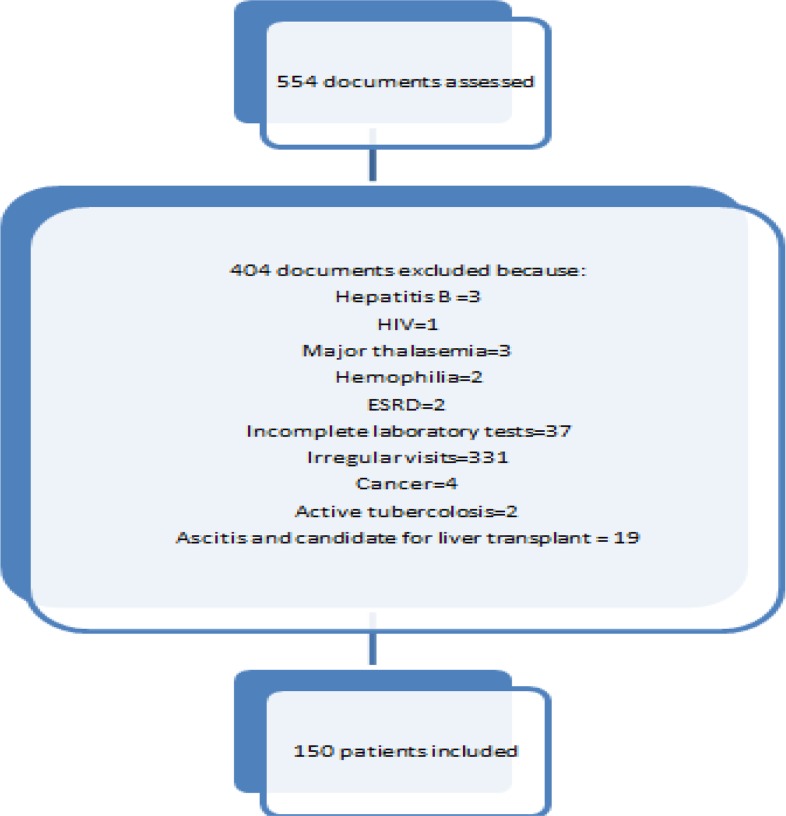
Diagram for the inclusion of the patients

In a cross-sectional study, all patients with positive hepatitis C referred to Hepatitis Clinic of Imam Khomeini Hospital Complex, Tehran, Iran, were eligible for treatment of chronic hepatitis from Apr 2009 to Mar 2014 after exclusion criteria applied. With convenient sampling, out of registered documents in our center, 554 patients were assessed and 150 patients were included (Fig. 1).

The study was approved ethically and methodologically by research council of infectious diseases department of Tehran University of Medical Sciences.

Hepatitis C viral load, complete blood count (CBC) and liver enzyme tests include: (AST, ALT, Bill, PT, INR) were measured before initiation of treatment. Duration of treatment was determined depending on the genotype of the virus from 6 months to 1 year.

During the treatment period, AST, ALT, and CBC rechecked monthly. CBC performed with automated analyzer.

All of patients were visiting every month and following of any new signs or symptoms, especially those related to infections such as fever, chills, cough, and dysuria by an infectious disease specialist.

### Inclusion criteria were

1. A definitive diagnosis of hepatitis C (positive HCV RNA PCR)
2. Regular patient visits to clinic (at least every two months)
3. Hematologic changes recorded on a regular basis (at least every two months)

### Exclusion criteria were

1. Other types of hepatitis (B, D) and HIV-1 infection
2. Autoimmune diseases, blood disorders (leukemia, lymphoma, hemophilia), renal diseases that need blood transfusion, symptomatic cardiovascular disease
3. Taking illegal drugs during treatment of hepatitis C
4. Treatment with bone marrow suppressive drugs in last six months

### Definitions

Anemia was defined as hemoglobin concentration 10–12 gr/dl (mild to moderate) and <10 gr/dl (severe).

Leukopenia was defined as WBC count 2000–4000 cells/ μL (mild to moderate), and <2000 cells/ μL (severe).

Thrombocytopenia was defined as platelet count < 150,000/ μL. This was further classified as mild to moderate (platelet count 100,000/ μL–150,000/ μL), and severe (<100,000/ μL) thrombocytopenia.

Laboratory tests such as complete blood cell count, bleeding associated with hematologic complications, infection secondary to neutropenia, the drug dose, and the rates of discontinuation were measured.

Independent variables including: age, sex, underlying disease (Such as diabetes mellitus, chronic renal failure without the need for treatment of anemia, chronic stable and asymptomatic heart and or lung disease), duration of HCV infection, and history of HCV treatment, liver transaminase levels, genotype and viral load of virus were assessed.

The supervisor assessed the accuracy of the collected data. Data were analyzed statistically by SPSS 16.0 (Chicago, IL, USA). The level of significance was considered at *P*≤0.05.

Maintenance of the information of all patients at all stages of the research was a priority. There was no cost for patients. All the states of the Helsinki principles were applied.

## Results

Out of 554 registered patients, 150 (135 male and 15 female) were included. Mean age was 39.7±10.7 (range 23–74) yr old. Inclusion chart of patients is shown in Fig. 1.

Five patients (3.3%) had previous history of treatment with Interferon. Thirteen patients (8.7%) had underlying diseases including diabetes mellitus (3 patients), hypothyroidism (3 patients), lichenplanus (2 patients) and others (5 cases).

Forty-six patients (30.7%) had genotype 1 and 99 (66%) genotypes 2 and 3 of hepatitis C virus. Genotyping was not done for 5 (3.3%) patients. Treatment regimens were prescribed as Pegafron+Ribavirin in 125 (83.3%), Interfron+Ribavirin 9 (6%) and Pegintron+Ribavirin in 16 (10.7%) of patients.

### Hematologic changes

**Fig. 2:**
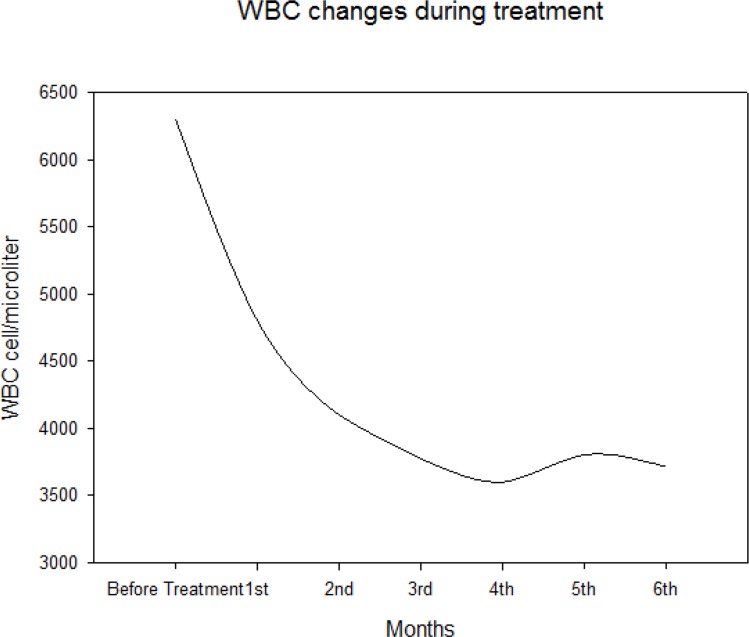
Hemoglobin changes during treatment

Out of 150 patients, hemoglobin concentrations of 72 (48%) patients were in normal ranges. Median of hemoglobin concentration was 15.3 gr/dl (IQR: 14.1–16.3) before initiation of treatment, it decreased to 13.95 gr/dl (IQR: 12.6–15.1) after one month and 12.42 (IQR:11.6–13.2) after 6 months of treatment (Fig. 2). Frequency of Hematologic changes according to gender; underlying disease, treatment regimen, genotype, age and viral load are shown in Table 1.

**Table 1:** Freq. of hematologic changes according to gender, underlying disease, treatment regimen, genotype, age and viral load

	*Group*	*Gender male N (%)*	*Underlying disease Yes* *N=13 n(%)*	*Treatment regimen*			*HCV Genotype*		*Age mean (SD)*	*Viral load*
				*Pegafron+Rib* *N=125*	*Interfron+Rib* *N=9*	*Pegintron+Rib* *N=16*	*1 N=46*	*2 and 3* *N=99*		
Hb changes	>12 mg/dl	70 (51.9)	2(15.4)	58(46.8)	5 (55.6)	8(50)	16(34.8)	54(54.5)	35.8(7.1)	2915849(6333916.8)
	10 to 12 mg/dl	51 (37.8)	7(53.8)	51(41.1)	4(44.4)	6(37.5)	22(47.8)	38(38.4)	41.2(11.6)	2312805(3969429.2)
	<10 mg/dl	14 (10.3)	4(30.8)	15(12.1)	0(0)	2(12.5)	8(17.4)	7(7.1)	51.3(11.7)	878154.3(1513051.3)
	***P*-value**	**0.018**	**0.014**		**0.931**		**0.044**		**<0.001**	**0.349**
WBC changes	>4000	30(21.6)	1 (7.7)	29(22.6)	3(33.3)	2(12.5)	7(15.2)	27(27.3)	35.7(9.3)	1335053(3030322.09)
	2000 to 4000	92(68.7)	12(92.3)	82(66.1)	6(66.7)	12(75)	34(73.9)	63(63.6)	40.3(11)	2996404(5801847.4)
	<4000	13(9.7)	0(0)	14(11.3)	0(0)	2(12.5)	5(10.9)	9(9.1)	45.1(9.8)	1026133(1944742.6)
	***P*-value**	**0.337**	**0.15**		**0.68**		**0.28**		**0.028**	**0.0161**
Platelet changes	>150000	58(42.5)	7(53.8)	52(41.1)	7(77.8)	5(31.3)	18(39.1)	46(46.5)	36.8(10.3)	1847576(3649004.8)
	100000 to 150000	48(35.8)	5(38.5)	44(35.5)	1(11.1)	7(43.8)	18(39.1)	34(34.3)	39.5(8.9)	3124755(6771816.4)
	<100000	29(21.6)	1(7.7)	29(23.4)	1(11.1)	4(25)	10(21.7)	19(19.2)	45.4(11.7)	2431803(4007733.02)
	***P*-value**	**0.571**	**0.487**		**0.338**		**0.71**		**0.02**	**0.418**

Anemia in women was more severe than men. The age of patients with severe anemia was older than patients with mild anemia or normal hemoglobin. The prevalence of anemia in people with underlying disease was higher than those without underlying disease. A significant relation between history of previous treatment and anemia was not observed. The prevalence of anemia in patients with genotype 1 virus was higher (*P*=0.044). No relation between the type of treatment and hemoglobin changes were observed. However, there was no association between sex and leukocyte changes, the changes in leukocyte count were higher in older patients. Moreover, we found an association between leukocyte count and the history of previous treatment.

Genotype of the virus and the type of treatment had no association with leukocyte changes.

**Fig. 3:**
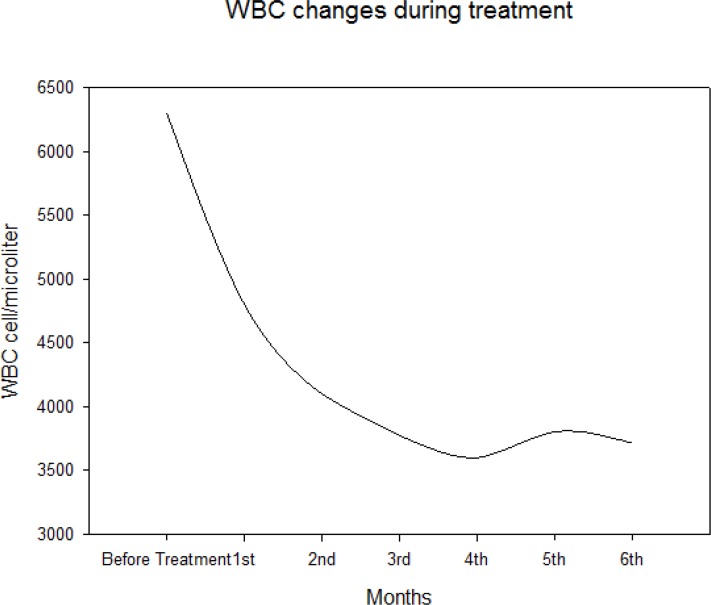
WBC changes during treatment

Median of WBC count was 6300 cell/microL (IQR: 4200–7787.5) before initiation of treatment, it decreased to 4800 cell/microL (IQR: 3515–5950) after one month and 3717.22 cell/microL (IQR: 3525–4100) after 6 months of treatment (Fig. 3).

Leukopenia is not significantly associated with serious infection. In our study, we just had one case of inguinal abscess that refers to surgeon for drainage; the patient had a previous history of splenectomy.

Due to the unavailability of differentiation count results for some of our patients, we could not report the number of absolute neutrophils counts (ANC).

**Fig. 4:**
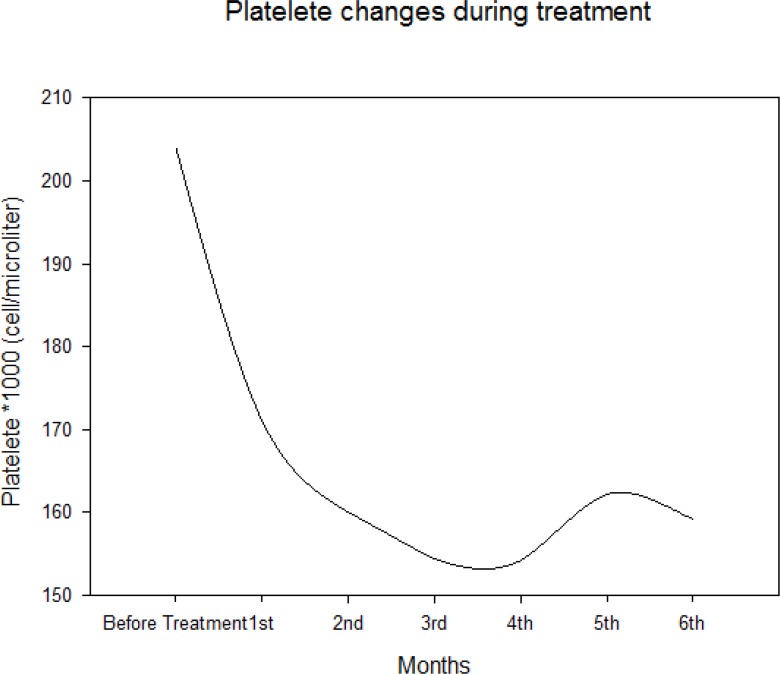
Platelet changes during treatment

Median of platelet count was 204000 cell/microL (IQR: 162250–238750) before initiation of treatment, it decreased to 171000 cell/microL (IQR: 141000–220750) after one month and 159150 cell/microL (IQR: 135250–188000) after 6 months of treatment (Fig. 4).

Fifty-two (34.6%) patients had mild and 34 (22.7%) had moderate and severe thrombocytopenia.

Older age and history of previous treatment for hepatitis C had association with thrombocytopenia. All of the patients with previous history of treatment catch thrombocytopenia. However, there was no association between the genotype of virus or treatment regimen with the count of platelet. Drug dosage was changed in 9 (6%) patients because of hematologic side effects. Six patients had severe anemia and Ribavirin dose was adjusted.

One patient had thrombocytopenia and two other patients had neutropenia then dose of interferon was adjusted.

One patient had platelet count lower than 25000 that was simultaneous with the end of treatment period.

Drug dose was changed in nine (6%) patients because of hematologic side effects (six cases of anemia, one case of thrombocytopenia and two cases of neutropenia). Frequency of other adverse effects and actions are showed in Table 2.

**Table 2:** Frequency of non-hematologictreatment related side effects and actions

***Adverse events***	***n (%) N=150***	***Actions***
Weakness	11(7.3)	Symptomatic therapy
Syncope	-	-
Cough and sputom	2 (1.3)	Assesment for infections were negative
Inguinal abscess	1(0.6)	Surgical drainage
Dental abcess(without fever)	1(0.6)	Temporary discontinuation of treatment
Epistaxis	1(0.6)	Symptomatic therapy
GI bleeding	2 (1.3)	Admission and treatment
Vaginal bleeding	1(0.6)	Symptomatic therapy
Itching	4(2.6)	Symptomatic therapy
Itching with skin lesions	1(0.6)	discontinuation of treatment
Erythrodermia	1(0.6)	discontinuation of treatment
Facial nerve palsy	1(0.6)	discontinuation of treatment
Resistant ascites	1(0.6)	discontinuation of treatment
Total	27 (100)	-

## Discussion

We found a low rate (6%) of drug dose adjustment due to hematologic changes in spite of relatively medium to high rate of anemia, leukopenia, and thrombocytopenia among 150 patients evaluated for hematological changes of hepatitis C treatment. In our study, 90% of patients were male. In other studies, the number of male patients was higher than women too (6–8).

Female gender due to several factors has a lower risk of chronic HCV infection. Allele specific gene IL28B, enhance the ability of infected person to clear hepatitis C virus from the body (9, 10). Moreover, mononuclear cells in females produce higher amount of INF-a by TLR7 stimulation thus can cause stronger innate immunity against viral infections (11).

The higher prevalence of risky behavior such as intravenous drug use among males in most regions including in our country is another explanation for higher prevalence of HCV infection in males. Therefore, based on the information it is expected to find the most cases of chronic hepatitis among men.

The mean age of the patients in our study was about 39 yr. In other studies, most of patients were in the third to fifth decades of life and the mean age was 33 to 54 yr (6, 8).

In all studies, including our study, younger age could be a warning to policy makers and health authorities. Because the age of high risk behavior has declined in recent decades.

In treatment of chronic hepatitis C patients, combination of interferon or PEGylated interferon (PEG-IFN) plus ribavirin is the recommended form of therapy because of availability, efficacy and also costs of treatment (12, 13). This combination has some hematologic side effects, like anemia, leukopenia, and thrombocytopenia.

### Anemia

The combination therapy was associated with hematological disorders. Anemia was the most important side effect because it can reduce health-related quality of life. Patients with anemia have symptoms like weakness and fatigue that affect their function. Anemia is one of the main reasons for change in dosage or discontinuation of treatment (14). In our study, anemia had the greatest role in changing the dose of drugs.

Interferon has inhibitory effects on bone marrow, and reduced levels of blood cells, including the count of RBCs. Moreover, Ribavirin can cause RBC lysis and enhances the inhibitory effect of interferon (15). Therefore, the combination of these two drugs has synergistic effects and decrease hemoglobin concentration. This anemia is called mixed anemia (16–18).

We changed the treatment regimen in 6 (4%) patients, but in previous studies, the frequency was higher and reported from 7% to 31% (12, 16).

The incidence of anemia in non-PEGylated IFN-treated patients is higher than the PEG-IFN-a (19). Ribavirin doses greater than 800mg/day, especially higher than 1000 mg/day is accompanied by higher anemia rate. However, we did not find any significant relationship between anemia and type of treatment. Usually, hemoglobin level drops at 4–8 wk of treatment and then remained at a plateau state and after end of treatment; it will cure (14). We found older age, female gender, having underlying diseases and genotype 1 HCV infection, as risk factors for anemia.

### Neutropenia

The most common side effect of combination therapy with Peg-IFNα and Ribavirin was severe neutropenia in a randomized control trial and these results were found in other studies (5, 6, 20). In a clinical trial on patients with HCV, neutropenia is the most common reason for dose reduction of PEG-INF in combination therapy regimens (14). Peg-IFN compared to the standard-IFNα has a higher risk for neutropenia and neutropenic risk may be dose-dependent (12).

In our study, frequency of severing leukopenia was 10.7% that is higher than previous reports. We did not consider to the WBC count before initiation of treatment, and this item could affect our findings. Use of Peg-IFNα and Ribavirin in most of our study patients may consider as a reason for higher prevalence of neutropenia in our study. However, like other studies, we found that leukopenia is not significantly associated with serious infection. Older age and positive history of previous treatment were two risk factors of neutropenia in our patients.

### Thrombocytopenia

Thrombocytopenia is one of side effects of interferon. Ibocytopenia in hepatitis C patients can happen; therefore, about 22% of patients with thrombocytopenia are seropositive for HCV, as reported in a previous study (21).

We found that older patients more likely to have thrombocytopenia. Platelet count lesser than 50000/ml was found in patients that have platelet count lesser than 100000/ml before the initiation of treatment. Similar results were found (22).

Dosage of PEG-IFN a-2b plus Rib can be changed because of severe thrombocytopenia (23). Drug dosage was changed just in one of our patients (0.6%) because of thrombocytopenia.

## Conclusion

The hematological changes are common side effects of conventional hepatitis C treatment regiments. Although drug dose adjustment is not usually necessary, due to severe anemia in older patients and patients with genotype 1, we should treat high-risk patients cautiously and made the appropriate changes in drug dosage at the right time.

## Ethical considerations

Ethical issues (Including plagiarism, informed consent, misconduct, data fabrication and/or falsification, double publication and/or submission, redundancy, etc.) have been completely observed by the authors.
